# Cryogenic X-Ray Microtomography of Early-Stage Polyurethane Foaming: 3D Analysis of Cell Structure Development

**DOI:** 10.3390/polym18020245

**Published:** 2026-01-16

**Authors:** Paula Cimavilla-Román, Suset Barroso-Solares, Mercedes Santiago-Calvo, Miguel Angel Rodriguez-Perez

**Affiliations:** 1 Cellular Materials Laboratory (CellMat), Condensed Matter Physics Department, University of Valladolid, 47011 Valladolid, Spain; suset.barroso@uva.es (S.B.-S.); mercesc88@gmail.com (M.S.-C.); marrod@uva.es (M.A.R.-P.); 2 BioEcoUva, Research Institute on Bioeconomy, University of Valladolid, 47011 Valladolid, Spain; 3 Study, Preservation, and Recovery of Archaeological, Historical and Environmental Heritage (AHMat) Research Group, Condensed Matter Physics, Crystallography, and Mineralogy Department, Faculty of Science, University of Valladolid, 47011 Valladolid, Spain

**Keywords:** polyurethane foam, X-ray tomography, cellular structure, foaming

## Abstract

Laboratory-scale cryogenic X-ray microtomography was employed for the first time to investigate the early structural evolution of polyurethane (PU) foams. This method enables ex situ studying the internal morphology of the frozen reactive mixture at various times before cell impingement. In this work, the precision of the method was evaluated by studying the early bubble formation and growth under different blowing agents and catalyst contents. It was detected that tripling the catalyst weight content doubled cell nucleation density, from 8.9 × 10^5^ to 1.8 × 10^6^ cells cm^−3^. Yet, doubling the water content has lesser impact on nucleation but leads to fast speeds of cell growth and, in turn, lower relative density at equal reaction times. Overall, it is demonstrated that laboratory cryogenic microtomography can be used to democratise the 3D investigation of the internal structure of foams which was until now only possible in synchrotron facilities. In addition, this method can help elucidate the mechanisms of nucleation and degeneration via directly measuring the density of bubbles and distance between them in the reactive mixture. Finally, this methodology could be extended to recent laboratory nanotomography systems utilizing X-ray tubes with nanometric spot sizes, thereby enabling the confident identification of nucleation events.

## 1. Introduction

The foaming of thermosetting polyurethane (PU) foams is a complex process in which the generation of a cellular structure is simultaneous to the polymer morphology build-up [1]. At the centre of the formation of rigid polyurethane (PU) foams is the reaction of isocyanates with compounds containing active hydrogen, in particular polyols and water. The reaction of isocyanate with polyol (polymerisation or gelling reaction) leads to the generation of urethane crosslinks, which build the polymer morphology [2], whereas the reaction of isocyanate with water (blowing or foaming reaction) is responsible for the generation of unstable carbamic acid, which spontaneously decomposes releasing amine and CO_2_ gas as a by-product [3]. According to classical nucleation theories, the released CO_2_ gas is responsible for saturating the liquid reactive mixture with gas. This phenomenon results in the nucleation of cells in the mixture and later blowing of the cells [4,5,6,7]. Yet, there are still open questions about the actual process of nucleation in PU foams. Some authors argue that supersaturation with CO_2_ gas is not achieved in the reactive mixture and thus the dissolved gas does not spontaneously create nuclei [4,8]. In contrast, the mechanical agitation needed to trigger foam formation constitutes a well-known source of air entrapments in the mixture [9,10,11]. The entrapped microbubbles can act as seeds for nucleation, hindering self-nucleation by the dissolved CO_2_ molecules [8,11,12]. Hence, in most cases, CO_2_ diffusion into the entrapped air bubbles proceeds so quickly that the nucleation of new bubbles is prevented, making it necessary to develop experimental approaches capable of identifying this phenomenon.

Due to the complexity of this foaming process, in recent years, much work has been carried out to develop techniques capable of probing the cellular structure formation in PU foams [13]. Among them, sequential X-ray imaging (X-ray radioscopy) has been widely used to investigate bubble nucleation and growth in PU foams produced using different types of nanoparticles as nucleating agents [14]. However, this technique has several drawbacks as it only provides 2D information, the sample must have reduced thickness, and the technique can hardly resolve the early stages of reaction (<60 s). For this reason, the technique does not permit characterising the cellular structure development near the instance of nucleation. In fact, detection of nucleation sites or following the growth of individual cells is difficult when using X-ray radioscopy. In a later study, Pérez-Tamarit et al. [15] used synchrotron X-ray tomography to investigate the microstructure generation in nanocomposite PU foams. Thanks to the advances in synchrotron facilities, the authors could acquire X-ray tomography scans at extremely fast speeds. In conventional tomography set-ups, acquisition can take up to 1 h, whereas using fast tomography a scan could be acquired in only 156 ms at a resolution of 3.2 µm [15]. The high temporal resolution provides 3D information on the cellular structure generated in PU foams filled with and without nanoparticles. From a scientific point of view, this is an ideal approach to studying the foaming behaviour of PU systems, but it is virtually impossible to reproduce such results with laboratory set-ups. In a follow-up to previous work, Reignier et al. [8] proved the potential of cryogenic scanning electron microscopy (cryo-SEM) to ex situ image the cellular structure development of PU foams. The high resolution of SEM enabled the visualisation of the structure on the nanometric scale. The authors focused on studying the structure at short reaction times. Using cryo-SEM they visualised the cavities generated by vaporised isopentane droplets as well as the morphology of the entrapped air bubbles introduced while mixing the reactants. The two structures revealed a drastically different morphology. Isopentane cavities had nanometric sizes and their number density was close to 10^12^ droplets cm^−3^. However, air bubbles had micrometric sizes (5–100 µm) and their number density was much lower, ca. 10^6^ cells cm^−3^, which is in the same order of magnitude as the final cell density in the cured foam. The authors then claimed that classical nucleation theory does not apply for PU foams prepared in open air containers [8]. The potential of cryo-SEM to ex situ visualise the early foaming of PU has been evidenced by follow-up studies [12] seeking to understand the role of perfluorocarbons. While this technique provides nanometric resolution essential to elucidate nascent nuclei, its broad application is limited due to its cost and availability [13]. In addition, from a scientific and statistical viewpoint, its accuracy is limited by the small sample size and by the fact that it can only render 2D information.

Considering the state of the art, in the present study, we make a novel contribution to the portfolio of techniques allowing the investigation of structure generation during PU foaming. Here, we present a new approach based on laboratory X-ray tomography to ex situ explore in 3D the cellular structure evolution. To this end, a cryogenic X-ray tomography set-up has been developed in which the samples are maintained at −120 °C during the scan. To the best of our knowledge, this is the first time that the evolution of PU foams has been studied in 3D by means of cryogenic tomography. This approach allows a detailed and statistically sound analysis of the evolution of the cellular structure and of the solid phase in 3D during the first stages of the foaming process.

## 2. Materials and Methods

### 2.1. Raw Materials of the Composite PU Foams

The polyol used was a high-functionality polyether polyol from Repsol S. A, Madrid, Spain, Alcupol R4520 (functionality: 4.5; OH value: 455 mg·KOH g^−1^; viscosity: 5250 mPa s at 25 °C). The Isocyanate was a polymeric diphenylmethane diisocyanate (pMDI), IsoPMDI 92,140 (31.5% NCO, density 1.23 g cm^−3^, viscosity 170-250 mPa s at 25 °C) supplied by BASF, Ludwigshafen, Germany. As gelling catalyst, Polycat 8 (N,N-dimethyl cyclohexylamine) from Evonik, Essen, Germany, was used. It is a tertiary amine of low water solubility which promotes the gelling reaction. TEGOSTAB^®^ B8522 (a non-hydrolysable polyether-polydimethylsiloxane–stabiliser) also from Evonik was used as a surfactant to obtain superior cell structures. The blowing agent was distilled water.

### 2.2. Preparation of PU Foams

In this study, we selected three formulations which undergo very different foaming kinetics and have been thoroughly studied in previous work by the authors if [16,17], so that we can evaluate the methodology’s sensitivity. More specifically, the components and weights to prepare 40 g foam can be found in Table 1. We designed three PU formulas: a control or reference formula (reference, REF), a formula with higher content of blowing agent, water in this case (blowing agent foam, BAF), and a formula with higher content of gelling catalyst versus the control foam (gelling catalyst foam, GCF) [17]. The isocyanate index was maintained constant for all foams at 110. Likewise, polyol and surfactant contents in parts per weight (ppw) were kept constant. Yet, the concentration of the catalyst and the blowing agent was changed to observe their impact on the early structural development. The selected concentrations were 0.5 and 1.5 ppw of catalyst and 2 and 5 ppw of water.

All components were blended together using an overhead stirrer (EUROSTAR 60 control from IKA, Barcelona, Spain), connected to a 50 mm diameter Vollrath Lenart-disc. First, a blend of polyol with additives (catalyst, surfactant, and blowing agent) was prepared by mixing at 250 rpm for 2 min. Afterwards, the PU foam reaction was triggered when mixing a total mass of 40 g, including the isocyanate and polyol blend, for 10 s at 1200 rpm in a 1 L plastic cup. The start of the stirring process between polyol blend and isocyanate is considered as time 0 of the reaction.

The parameters of the final cellular structure of these cured foams, previously reported, can be found in Table 2. It is important to note that the reported cell nucleation density might be lower than the actual value at the beginning of the foaming process, since it is calculated from the cured foam parameters, assuming that there are no degeneration mechanisms during foaming [18]. For this reason, this parameter is reported as estimated cell nucleation density, $N_{o}^{*}$ .

In these foams, the increase in the catalyst content only slightly decreases their density (Table 2). Conversely, density dropped approximately 1.6 times when the content of water rose from 2 to 5 ppw (Table 2). On the contrary, the increase in the gelling catalyst concentration significantly reduced the cell size by 23% for GCF with respect to REF (Table 2). Moreover, cell density decreased with the addition of water (Table 2). This change suggests an increase in the number of degeneration events during foaming. On the contrary, cell density rose with catalyst concentration. Similarly, estimated cell nucleation density increased by more than a factor 2 with the addition of more catalyst and only slightly with the addition of water (Table 2). Regarding anisotropy, all foams showed a structure with cells elongated in the foam growth direction (Table 2). Yet, anisotropy was higher for samples of lower density (BAF).

### 2.3. Sample Preparation and Freezing for Cryogenic X-Ray Tomography

To prepare the samples for the cryogenic X-ray tomography experiments, a few millilitres of the reactive mixture were extracted from the preparation cup (Section 2.2) right after stirring and injected into the tomography sample holder. The sample holder is a hollow cylindrical Kapton container of 4 mm in diameter and 2 cm in height. It can accommodate a maximum volume of 0.25 mL. In order to ensure reproducibility between experiments, the injected mixture volume was fixed to 0.1 mL, and then the liquid was allowed to expand in the container until the corresponding freezing time. The foam expansion was first stopped with the vapours of liquid nitrogen (LN_2_), which boil at −196 °C forming a visible fog with temperature often ranging from −190 °C to −150 °C. After one to two seconds, the sample is immersed in liquid nitrogen to achieve full freezing and storage. This two-step freezing protocol is essential to prevent collapse due to the sudden freezing of the blown reactive mixture. In this work, we define the foaming time as the time elapsed between the start of the mixing process and the time at which the samples were frozen with liquid nitrogen. Three PU formulations and three distinct foaming times for each were studied.

The subject of whether rapid freezing leads to changes in the internal structure has been previously discussed by authors using cryo-SEM [8,19]. Artefacts caused by freezing are typically structural collapse of the centre of the sample further away from the cryogenic medium, longitudinal stress fractures, or ice formation. Collapse was avoided by following the freezing protocol detailed in Section 2.3. Fractures were at times observed in the internal structure but due to their distinctive anisotropic shape, they were easily recognised and such samples were excluded from the analysis. The formation of ice crystal structures is not expected to influence the results of the work as their size (50 nm) is far below the typical resolution of microtomography set-up.

### 2.4. Cryogenic X-Ray Tomography

The tomography experiments were performed using a laboratory X-ray microtomography system. The set-up consisted of an X-ray microfocus source (Hamamatsu, Shizuoka, Japan) and a flat panel detector connected to a frame grabber (Dalsa-Coreco, CA, USA), which records the projection images. This high-resolution detector is composed of a matrix of 2240 × 2344 pixels, each with a size of 50 μm. In these experiments, the tube voltage was set to 55 kV and the tube current to 170 µA, the detector exposure time was 1 s, and each projection was the result of averaging three consecutive images to reduce noise. On the whole, 1200 projections were acquired, resulting in long scans of ca. 90 min. The spatial resolution was fixed to 3.5 μm.

**Figure 1:**
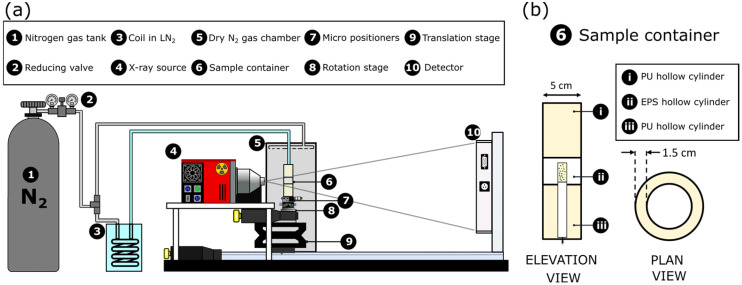
(**a**) Scheme of the cooling and X-ray tomography system. Nitrogen gas (1), at room temperature, passes through a reducing valve (2) to a coil (3) immersed in liquid nitrogen. The cooled gas enters the insulation container (6) through a tube. Both the sample and the container sit on top of the rotation stage (8) which is inside a dry gas chamber (5). (**b**) Elevation and plan view of the insulating container with sample superimposed on the elevation view.

The described microtomography set-up per se lacked the cooling system required to keep the samples at cryogenic temperatures during the scan. Hence, a cooling stage was implemented to ensure the dimensional stability of the foams during the inspection. Figure 1 shows a scheme of the X-ray cryo-tomography set-up.

The reactive sample (prepared as specified in Section 2.4) was located inside a thermal insulating container. This container was a hollow cylinder of 5 cm in diameter and walls of 1.5 cm in thickness (Figure 1b). The container was made up of a low-density foam for its high insulation capacity, namely, PU foam, and expanded polystyrene (EPS) foam in the region crossing the X-ray beam path. EPS is an ideal material in terms of X-ray transparency as it has extremely low density and low attenuation to the X-rays. In the X-ray tomography set-up, both the container and the sample stand on top of a rotation stage (Figure 1a(8)) so that the sample and container rotate synchronously with the stage.

Low temperatures in the sample are achieved by making dry nitrogen gas (N_2_), coming from a pressurised gas bottle, pass through a copper coil immersed in liquid nitrogen. After the gas is cooled, it is directed towards the X-ray set-up and into the sample container. In addition, as seen in Figure 1, the entire sample set-up (sample container and rotational stage) is enclosed inside a chamber filled with N_2_ gas at room temperature. As the chamber is not airtight, N_2_ gas coming from the tank is flowing continuously in and out of the chamber (Figure 1). The continuous flow of N_2_ gas is essential to prevent the formation of frost on the surface of the sample container. Avoiding the formation of frost is a critical factor when performing cryogenic X-ray tomography since the dynamic generation of frost would hinder the reconstruction of the acquired tomographies [20]. For this work, the operating pressure for both the cold N_2_ and the room temperature N_2_ was maintained at 1.5 bar. This pressure ensured that the temperature of the gas when reaching the sample was sufficiently low to keep the material frozen. The temperature inside the sample container (Figure 1, component 6) was measured with a type K thermocouple during the scan. The temperature was logged with a Data Logger from Picolog, and during the experiment time, the temperature was approximately −117.1 ± 7.4 °C. Average temperature was rather constant for every experiment, suffering random fluctuations within the error margins along the experiments due to experimental limitations of the in-house set-up. Nevertheless, we are confident that there was no influence of the temperature fluctuations in the frozen samples for two key facts. Firstly, temperature always stayed below 100 °C and, secondly, the sharpness of the pores in the reconstructions indicated that no changes inside the sample took place during the scan; otherwise, motion artefacts such as blurring or edge effects would have been observed [21].

### 2.5. Image Analysis

The reconstruction of the cryogenic X-ray tomographies was carried out using the Octopus reconstruction package [22]. The process produces slices or cross-section images of the scanned volumes. The analysis of the foam microstructure was conducted in at least four regions within each sample of volume near 5.5 mm^3^. These volumes were analysed using the ImageJ/FIJI software tool, version 1.53i [23]. The analysis focused on characterising the cellular structure (gas phase) and the thickness of the reactive mixture matrix (solid phase). The gas phase was studied using the MorphoLibJ plugin implemented on ImageJ/FIJI [24]. Firstly, an edge-preserving filter was applied to reduce noise in the images and enhance the contrast between the solid and gas phases. Afterwards, the gas phase was binarized to separate both phases. From the binarized volumes, cell size, ϕ, cell density, *N_v_*, relative density, ρr, and porosity, *V_f_*, were measured in 3D. Relative density and porosity were estimated simply as the number of solid and empty voxels, respectively, divided by the total number of voxels in the analysed volume. Cell size was obtained assuming that the cells had spherical shapes. Hence, the average cell size, Φ, is the average diameter of the cells. Moreover, the cell size distribution was obtained, and the cell size distribution homogeneity was evaluated by the normalised standard deviation, calculated as the ratio between the standard deviation of the distribution, SD, and the average cell size, Φ. Cell density, *N_v_,* was calculated by dividing the total number of cells by the volume of the analysed region. Employing Kumar’s method [25], the estimated cell nucleation density (Equation (1)) was calculated, $N_{o}^{*}$ . It should be noted that when studying early foaming stages, the estimated cell nucleation density should be rather accurate. For the analysis, more than 10^4^ cells were studied.

(1) $$N_{o}^{*}=\frac{N_{v}}{\left(1-V_{f}\right)}$$

After binarization, the thickness of the matrix (solid phase) was measured using a Local Thickness algorithm implemented on FIJI [26]. The plugin returns a thickness map of the binarized phase. From the thickness map, a histogram yielding the matrix thickness distribution was calculated and from it, the average histogram thickness was calculated, providing the intercell distance, δ.

## 3. Results

**Figure 2:**
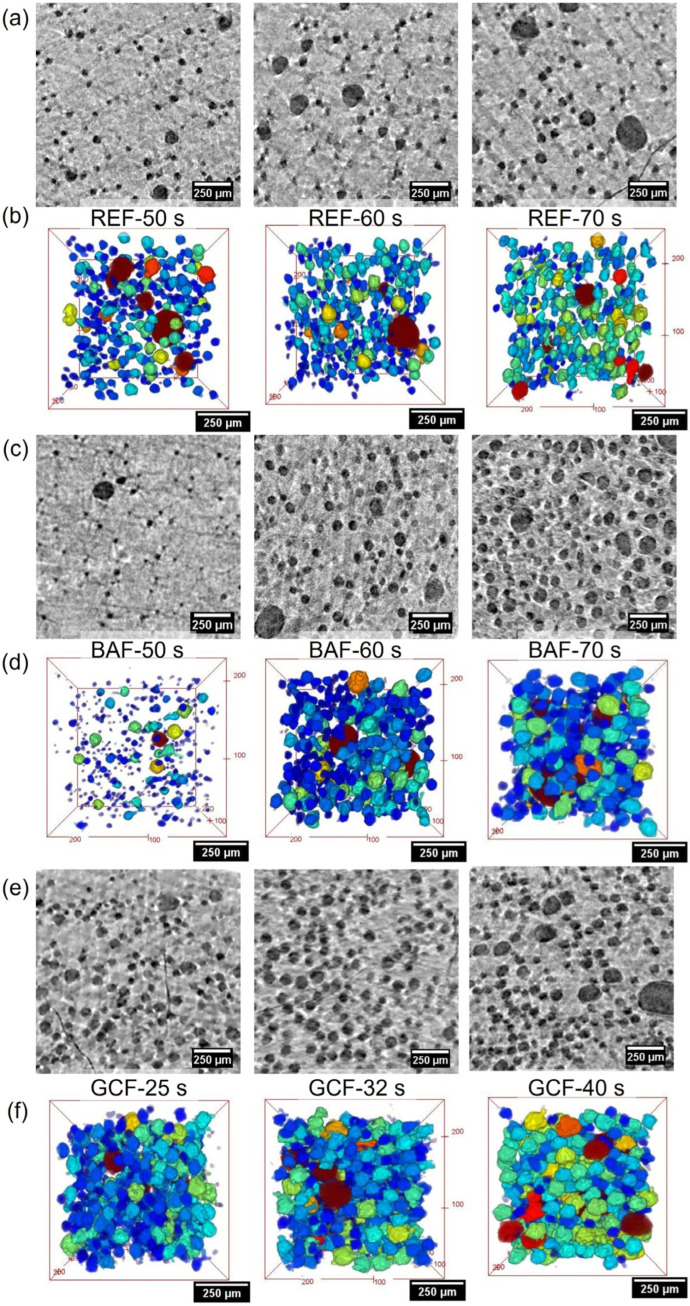
(**a**,**c**,**e**) 2D reconstructed cross-sections of the scanned samples in greyscale, light grey corresponds to the mixture while dark grey is the gas phase. (**b**,**d**,**f**) 3D renderings of the analysed cells for formulas REF, BAF, and GCF at different times. Renders are shown in rainbow colour scale in which cells coloured in dark blue correspond to the smallest cells (<25 µm) while orange and red indicate largest cell sizes (>100 µm).

The reactivity of these foams was studied in previous work [17]. It was detected that the time required for the cells to nucleate (cream or expansion time) decreased from 72 s for foam REF to 42 s for foam GCF. However, the increase in blowing agent only reduced slightly the cream time from 72 s (REF) to 54 s (BAF). To understand the early cellular structure development of PU foams, we analysed reaction times close to the cream time. For systems REF and BAF, the selected freezing times were 50, 60, and 70 s, whereas, for system GCF, due to its high reactivity, the selected freezing times were 25, 32, and 40 s. In Figure 2, the structure of all the frozen PU samples obtained by cryogenic X-ray tomography can be appreciated.

From Figure 2, it is possible to appreciate some qualitative differences between samples. For foam REF of low catalyst and water content (Figure 2a,b), the structure remains practically unchanged at the short foaming times considered, whereas foam BAF, with higher water content (Figure 2c,d), transitions in only 20 s from a structure with nascent cells of only a few microns into fully blown spherical bubbles a few microns apart from each other. Lastly, despite being examined at shorter reaction times, foam GCF (Figure 2d,e) revealed higher number of cells, porosity, and larger cells than the REF and BAF foams with lower catalyst concentration.

From the tomography volumes, the main descriptors of the cellular structure were measured (Table 3). The relative density results gathered in Table 3 evidence that all systems with low catalyst content (i.e., 0.5 ppw) undergo little gas generation before cream time. Before the onset of foam growth, the porosity of foams REF and BAF was below 10%. This fact is particularly noticeable in system REF, with cream time close to 72 s. This system reveals relatively low expansion and negligible structural changes in the characterised reaction times. Likewise, the cell growth experienced by foam REF is almost negligible (0.5 µm s^−1^) compared to typical cell growth rates (~3 µm s^−1^) observed, after the cream time when using X-ray radioscopy [16].

After the onset of foam expansion (54 s), BAF samples suffer a quick growth as anticipated by the images in Figure 2. In only 20 s, porosity increased to about 80%. Additionally, there is a drastic increase in cell size in less than 10 s (from 50 s to 60 s of reaction time). This translates into a speed of cell growth of approximately 2.6 µm s^−1^. The fast speed of cell growth is a consequence of the high content of water in the formulation. In PU foams, the blowing (urea) reaction proceeds at a faster pace than the polymerisation [27,28]. Hence, the CO_2_ gas generated as a by-product of the urea reaction quickly diffuses into the nuclei blowing the cells.

In contrast, the formulation with a higher catalyst content (GCF) undergoes quite a different structural development. Firstly, even at times lower than cream, the system has reached lower relative densities and larger cell sizes compared to foams REF and BAF. In the 15 s of monitored reaction time, the speed of cell growth was close to 1.2 µm s^−1^, less than half of that foam BAF. We attribute this lower growth speed to the fast viscosity build-up caused by the high reactivity of the foam more than to lower reaction speed [17].

Before deepening on the analysis of other descriptors of the cellular structure, it is necessary to address the potential presence of cell degeneration mechanisms on the studied foams. With this aim, the evolution of the matrix thickness is analysed.

**Figure 3:**
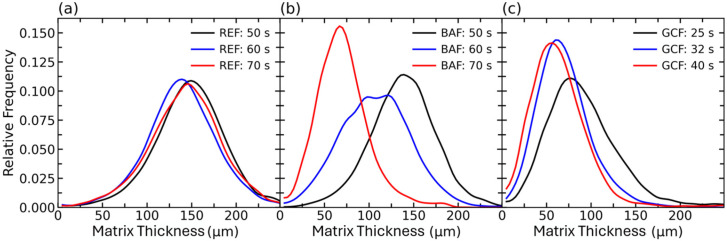
Thickness of the solid matrix in between cells at three different reaction times for systems (**a**) REF, (**b**) BAF, and (**c**) GCF.

From Figure 2 and Figure 3, it can be observed how the matrix thickness is clearly in the micrometric range. At high values of the relative density (*ρ_r_* > 0.9) foams REF and BAF show average intercell distance larger than 140 µm. However, for foam GCF, at a similar relative density ca. 0.9, the average intercell distance is below 100 due to the higher cell nucleation density of the foam. This high intercell distance suggests a negligible impact of degeneration mechanisms within the studied timeframe of the PU foams. It is known that in PU foams coalescence is one of the most common degeneration mechanisms, being triggered by the reduction in wall thickness below the micron [29,30]. However, during the times studied in this work, cells had spherical shapes and were separated from each other by more than 5 µm, as reported in Figure 3, making the appearance of coalescence events unlikely. For foam REF, the absence of significant expansion during the studied timeframe is also observed in the matrix thickness histogram Figure 3a. On the contrary, the noticeable blowing of cells in foam BAF result in a decrease of the solid matrix thickness from about 140 μm to 70 μm in only 20 s. Finally, the higher cell size and cell density in GCF foams result in a much lower initial solid matrix thickness of about 90 μm, while at longer times the solid matrix thickness is reduced to about 60–70 μm.

**Figure 4:**
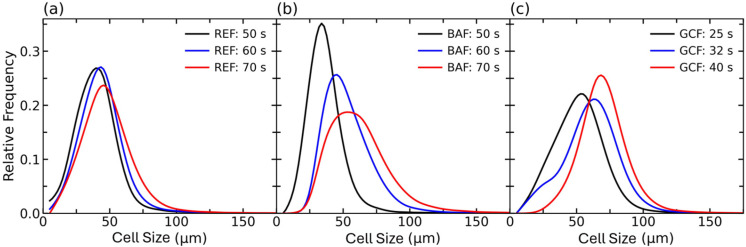
Cell size distributions for systems at three different reaction times for systems (**a**) REF, (**b**) BAF, and (**c**) GCF.

Then, after analysing a large number of cells, more than 10^4^ for each experiment, the cell size distributions (Figure 4), average cell size, cell densities, and estimated cell nucleation densities were calculated (Table 3).

In foam REF, cell distributions remain virtually unchanged in accordance with the average cell size and SD/Φ values (Table 3). In 20 s, only a slight displacement of the distribution to higher cell sizes is observed (Figure 4a), although small cells, near the scan spatial resolution, are still found after 70 s, evidencing the negligible evolution of this foam during the studied times. Accordingly, to the expected almost static situation before the cream time, the estimated cell nucleation density did not present significative changes during the studied timeframe (Table 3), being stable at ca. 9 × 10^5^ nuclei cm^−3^.

In contrast, system BAF at 50 s of reaction (Figure 4b) presents a very similar structure to that of foam REF. The structure shows cells with sizes as small as 8 µm. After 50 s of reaction the sample showed a fast cell growth with the minimum cell size growing above 20 µm. Moreover, the generation of gas due to the blowing reaction resulted in a change in cellular structure heterogeneity, as evidenced by the broadening of the cell size distribution. This increased heterogeneity is also reflected in the increase in the standard deviation reported in Table 3. Here, it is uncertain whether the increase in the heterogeneity of the cellular structure is due to the creation of new nuclei due to the ongoing gas generation or to an instrumental artefact due to the growth of nuclei towards detectable sizes above the spatial resolution (ca. 3.5 µm). Cellular structure heterogeneity is further evidenced by the slight increase in estimated cell nucleation from 50 to 70 s (Table 3). Last but not least, the increase in catalyst content (i.e., GCF foam) caused the cell distribution to move to higher cell sizes at early reaction times compared to the other foams. However, at 32 s of reaction, a population of small-sized cells (<20 µm) prevailed, with a cell size distribution suggesting a potential bimodal population, one centred at sizes over 50 μm and another one at sizes about 25 μm. This could be the result of nucleation happening within or near the studied timeframe, with the initial population of cells detected at 25 s shifting towards higher cell sizes, and another population of nuclei or cells below the limit of detection shifting towards detectable sizes at 32 s. Then, all the detected cells keep growing without new cells appearing. This interpretation is in good agreement with the slight increase in estimated cell nucleation density found between 25 and 32 s, and the similar values found at 32 and 40 s.

Therefore, while the estimated cell nucleation density remains constant for REF, it seems to increase over the monitored time for foams BAF and GCF. In principle, this points to an ongoing nucleation near the cream time for the latter foams. However, in previous studies [8,31] it has been argued that nucleation in PU foams is typically the result of air bubbles being entrapped during the mixing of reactants. Hence, another possible explanation for the apparent increase in cell nucleation density is the limited resolution (3.5 µm) of the X-ray system. The limited spatial resolution may hinder the visualisation of cells below or near the spatial resolution at early reaction times. However, once they have grown above the spatial resolution of the set up, they are detected and factored in the cell density count. Regardless of this fact, the results show that the foam with the highest cell nucleation density was the one with the highest catalyst content (GCF). This agrees with the final cellular structure of the cured foam (Table 2). It should be noted that the highest cell nucleation density found in the GCF foam could be a consequence of more efficient gas entrapment by the higher viscosity of the mixture [17].

Therefore, the presented experimental methodology has been proven to be a suitable pathway to investigate early stages of cellular structure development in 3D using laboratory X-ray sources. This method can bridge an existing technical gap in the study of the structure of reactive foams at short reaction times using lab-based X-ray instruments. Here, the estimated cell nucleation values, about 10^6^ nuclei cm^−3^, and initial micrometric cell sizes matched with the typical values for air bubbles proposed in recent cryo-SEM work [8,12]. Based on these experimental results, the classical nucleation theory is rejected in the case of free-rise PU foams due to the very high energy barrier required. It is more likely that air bubbles introduced during stirring act as nuclei for the foam structure. The CO_2_ gas generated in the blowing reaction simply blows the pre-existing nuclei rather than forming new ones. Hence, in our work and in previous microtomography reports, the apparent increase in cell nucleation density is most likely a consequence of the limited spatial resolution of the tomography set-up rather than to the generation of new cells [14,15]. In order to confidently confirm this hypothesis, high-resolution tomography should be performed on PU foams to observe bubbles and nuclei in the nanoscale.

## 4. Conclusions

In this work, the development of a new technique probing the fast-changing cellular structure of PU foams is reported. The proposed approach permits the ex situ investigation of the foaming process of PU and relies on cryogenic temperatures to vitrify the cellular structure of the foam at different reaction times. By employing an in-house cooling system, the dimensional stability of frozen PU samples was ensured, and their microstructure could be explored in 3D with laboratory X-ray tomography.

Thanks to this technique, the cellular structure of PU foams of different reactivity was studied near their cream time. For foams of low catalyst content, the cell size and intercell distance remained practically unchanged before the cream time, whereas after the cream time the cell size nearly doubled and the average distance between cells dropped. Initially, cells were isolated from each other by large solid regions as thick as 150 µm. Nonetheless, the intercell distance rapidly decreased with time and halved in only 20 s. For foams with high catalyst content (1.5 ppw), the cell growth and reduction in intercell distance started slightly before the cream time. In addition, the cell growth rate was slower than in foams with higher water content due to the fast viscosity build-up caused by the high reactivity of the foam. The cell nucleation density was also the highest for the foam with the highest catalyst content, most probably as a consequence of more efficient gas entrapment by the higher viscosity mixture.

The proposed experimental approach proved to be sensible to all these changes during the earliest stages of the PU foaming process. Therefore, future developments combining this approach with X-ray tomography of higher resolution (e.g., synchrotron facilities) could provide solid evidence about the role of air entrapments in the nucleation of traditional polyurethane foams and novel functionalized polyurethane systems [32].

## Figures and Tables

**Table 1:** PU formulation components and their corresponding weights.

Sample	Isocyanate (g)	Polyol (g)	Surfactant (g)	Gelling Catalyst (g)	Water (g)
REF	23.67	15.78	0.16	0.08	0.32
BAF	26.06	13.09	0.13	0.07	0.66
GCF	23.58	15.72	0.16	0.24	0.31

**Table 2:** Density, relative density, and main descriptors of the cellular structure of the cured foams obtained from SEM micrographs: foam density (*ρ*), relative density (*ρ_r_*), average cell size (*Φ*_3D_), anisotropy ratio of the cells in the foam rise direction (*AR*), cell density (*N_v_*), and estimated cell nucleation density ( $N_{o}^{*}$ ).

Sample	*ρ* (kg m^−3^)	$\rho_{r}$	*Φ*_3D_ (µm)	*AR*	*N_v_* (Cells·cm^−3^)	$N_{o}^{*}$ (Cells·cm^−3^)
REF	59.7 ± 1.6	0.051	416.0 ± 91.8	1.2	2.5 × 10^4^	4.5 × 10^5^
BAF	34.4 ± 0.2	0.030	488.3 ± 151.9	1.4	1.6 × 10^4^	4.8 × 10^5^
GCF	52.3 ± 1.1	0.045	322.5 ± 73.7	1.3	5.4 × 10^4^	1.1 × 10^6^

**Table 3:** Evolution of the main structural descriptors near the cream time for the foams in Table 1.

Sample	Reaction Time (s)	*ρ_r_*	*φ* (µm)	*SD/φ*	*N_v_* (Cells·cm^−3^)	$N_{o}^{*}$ (Cells·cm^−3^)	*δ* (µm)
REF	50	0.93 ± 0.01	39.5 ± 18.1	0.46	8.1 ± 0.6 × 10^5^	8.7 ± 0.6 × 10^5^	148.4 ± 40.9
	60	0.91 ± 0.02	43.6 ± 20.3	0.47	8.5 ± 0.5 × 10^5^	9.3 ± 0.5 × 10^5^	139.9 ± 41.0
	70	0.91 ± 0.02	49.3 ± 20.7	0.42	8.0 ± 0.7 × 10^5^	8.9 ± 0.8 × 10^5^	143.6 ± 42.6
BAF	50	0.96 ± 0.01	31.2 ± 13.5	0.43	1.1 ± 0.1 × 10^6^	1.2 ± 0.1 × 10^6^	138.8 ± 38.9
	60	0.90 ± 0.02	56.4 ± 20.3	0.36	1.0 ± 0.4 × 10^6^	1.1 ± 0.4 × 10^6^	106.9 ± 39.1
	70	0.77 ± 0.02	62.9 ± 24.5	0.39	1.1 ± 0.1 × 10^6^	1.4 ± 0.1 × 10^6^	70.5 ± 29.0
GCF	25	0.87 ± 0.01	51.9 ± 18.1	0.35	1.3 ± 0.1 × 10^6^	1.5 ± 0.1 × 10^6^	93.9 ± 47.5
	32	0.78 ± 0.03	60.3 ± 20.7	0.34	1.3 ± 0.2 × 10^6^	1.7 ± 0.3 × 10^6^	69.9 ± 30.6
	40	0.71 ± 0.05	70.5 ± 18.5	0.26	1.3 ± 0.2 × 10^6^	1.8 ± 0.3 × 10^6^	63.6 ± 31.3

## Data Availability

The original contributions presented in this study are included in the article. Further inquiries can be directed to the corresponding author.
